# Single Near‐Infrared Emissive Polymer Nanoparticles as Versatile Phototheranostics

**DOI:** 10.1002/advs.201700085

**Published:** 2017-06-10

**Authors:** Liang Guo, Guangle Niu, Xiuli Zheng, Jiechao Ge, Weimin Liu, Qingyan Jia, Panpan Zhang, Hongyan Zhang, Pengfei Wang

**Affiliations:** ^1^ Key Laboratory of Photochemical Conversion and Optoelectronic Materials and CityU‐CAS Joint Laboratory of Functional Materials and Devices Technical Institute of Physics and Chemistry Chinese Academy of Sciences Beijing 100190 China; ^2^ School of Future Technology University of Chinese Academy of Sciences Beijing 100049 China

**Keywords:** fluorescence imaging, photoacoustic imaging, photodynamic therapy, photothermal therapy, phototheranostics, polymer nanoparticles

## Abstract

Attaining consistently high performance of diagnostic and therapeutic functions in one single nanoplatform is of great significance for nanomedicine. This study demonstrates the use of donor–acceptor (D–A) structured polymer (TBT) to develop a smart “all‐five‐in‐one” theranostic that conveniently integrates fluorescence/photoacoustic/thermal imaging and photodynamic/photothermal therapy into single nanoparticle. The prepared nanoparticles (TBTPNPs) exhibit near‐infrared emission, high water solubility, excellent light resistance, good pH stability, and negligible toxicity. Additionally, the TBTPNPs exhibit an excellent singlet oxygen (^1^O_2_) quantum yield (40%) and high photothermal conversion efficiency (37.1%) under single‐laser irradiation (635 nm). Apart from their two phototherapeutic modalities, fluorescence, photoacoustic signals, and thermal imaging in vivo can be simultaneously achieved because of their enhanced permeability and retention effects. This work demonstrates that the prepared TBTPNPs are “all‐five‐in‐one” phototheranostic agents that can exhibit properties to satisfy the “one‐fits‐all” requirement for future phototheranostic applications. Thus, the prepared TBTPNPs can provide fundamental insights into the development of PNP‐based nanoagents for cancer therapy.

## 1 Introduction

Phototheranostic applications have attracted worldwide attention in recent years because they can enable precision treatment and exhibit high therapeutic effects by combining therapy with light‐triggered diagnosis and using various optical imaging techniques.^1^ Among the various imaging techniques, fluorescence (FL) and photoacoustic (PA) imaging have intrinsic advantages, such as good sensitivity, spatial resolution, and imaging depth.^2^ However, despite the high temporal resolution and sensitivity of FL imaging, it is inefficient for in vivo applications mainly because of its limited spatial resolution. PA imaging is a whole‐body imaging modality that offers high spatial resolution, deep penetration, and high contrast in vivo.^3^ Therefore, designing nanomaterials that combine FL and PA imaging modalities in a single agent would exploit the advantages of these techniques while overcoming the respective disadvantages of each technique, that is, such nanomaterials could maintain imaging sensitivity while exhibiting high spatial resolution and deep tissue penetration, which are all advantageous properties for tumor detection.^4^ Apart from light‐triggered imaging, light‐induced therapeutics, such as photodynamic therapy (PDT) and photothermal therapy (PTT), are regarded as promising and safe techniques because of their unique advantages, such as minimal invasion, high selectivity, and low recurrence probability.^5^ In PDT and PTT, light is absorbed through a photosensitizer that then generates reactive oxygen species (ROS) or heat, to kill cancer cells.^6^ However, cancer cells rapidly grow and have distorted tumor blood vessels, which all restrict oxygen supply to the tumor site, thus leading to ineffective PDT treatment.^7^ For this reason, many hyperthermia agents, such as gold‐,^8^ carbon‐,^9^ and organic‐10 based nanostructures, have been developed to overcome the limitations of PDT and improve the therapeutic efficiency. These agents are prepared as carriers through encapsulating or conjugating the PDT agents to enable simultaneous PDT and PTT treatment. However, in this approach, some potential problems remain, including unstable nanostructures, altered surfaces, and increasing pharmacokinetic complexity.^11^ Therefore, developing phototheranostic agents that originate from single versatile and efficient nanoparticles that are applicable for both multimodal imaging as well as phototherapy is of great importance.

Polymer nanoparticles (PNPs) are newly developed optical nanomaterials that have attracted widespread attention because they have large extinction coefficients, exceptional fluorescence brightness, excellent photostability, and good biocompatibility.^12^ Owing to these properties, PNPs have potential uses in the field of biosensors,^13^ bioimaging,^14^ and phototherapy.^15^ In addition, PNPs have been extensively researched for phototheranostic applications.^16^ However, although some impressive advances have been achieved for PNP‐based phototheranostics, particularly in PA‐imaging‐guided PTT or FL‐imaging‐guided PDT,^17^ reports on multifunctional and single‐PNP‐based phototheranostic applications are few, and thus, satisfying the “one‐fits‐all” requirement for generalized phototheranostics remains challenging.

**Scheme 1:**
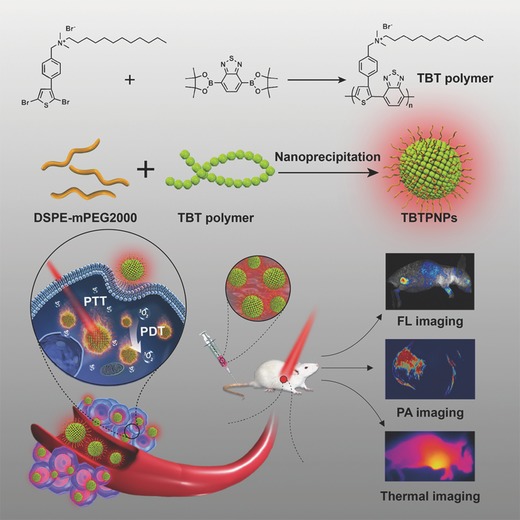
Structure of polymer TBT and five‐in‐one functionalized TBTPNPs for FL, PA, thermal imaging, and PDT/PTT.

Previously, our group synthesized versatile PNPs based on polythiophene (PT2) as two‐photon‐excited photosensitizers to localize lysosomes for simultaneous cellular and deep‐tissue imaging and PDT.^18^ However, the as‐prepared PNPs exhibited short wavelength absorption (absorption peak of 410 nm) and fluorescence (emission peak of 590 nm) in water. Therefore, developing PNPs with long wavelength absorption and emission, especially in the near‐infrared (NIR) region, is of great importance for in vivo applications. Benzo[c][1,2,5]thiadiazole (BT), a strong electron‐withdrawing unit, has been widely used for the preparation of materials that are based on donor–acceptor (D–A) structures and exhibit long wavelength absorption or emission for bioimaging and electronic device applications.^19^ Based on these previous results, here, we designed and synthesized a thiophene–BT‐based D–A polymer (TBT, **Scheme** **1**) with strong red shifted absorption and emission. Then, we prepared TBTPNPs using 2‐distearoyl‐sn‐glycero‐3‐phosphoethanolamine‐N‐[methoxy‐(polyethyleneglycol)‐2000] (DSPE‐mPEG2000) as an encapsulation matrix via one‐step nanoprecipitation (Scheme 1). The obtained TBTPNPs exhibited broad absorption (400–750 nm), NIR emission (an FL peak of 690 nm), high water solubility, excellent light resistance, and negligible toxicity. Notably, the prepared TBTPNPs simultaneously showed PDT and PTT effects under 635 nm laser irradiation (≈40% singlet oxygen (^1^O_2_) quantum yield and ≈37.1% photothermal conversion efficiency, respectively). Apart from the two phototherapeutic modalities, FL, PA signal, and thermal imaging using TBTPNPs can be simultaneously achieved. Our in vitro and in vivo studies indicated that the as‐prepared TBTPNPs are versatile phototheranostic agents for tri‐modal imaging‐guided simultaneous PDT and PTT. As far as we know, our study is the first to investigate “all‐five‐in‐one” phototheranostics based on a single type of PNP.

## 2 Results and Discussion

### 2.1 Synthesis and Characterization of TBTPNPs

**Figure 1:**
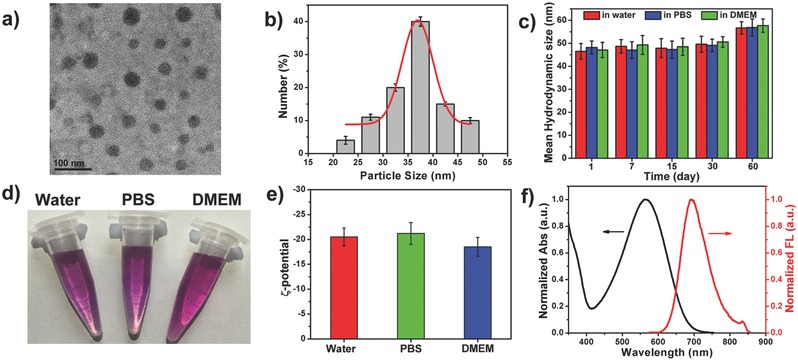
a) TEM picture of TBTPNPs. b) Size distribution of TBTPNPs. c) DLS of TBTPNPs in water, PBS, and DMEM for 60 d. d) Photograph of TBTPNPs dissolved in water (left), PBS (middle), and DMEM (right). e) ζ‐potential of TBTPNPs in water, PBS, and DMEM. f) Normalized absorption and fluorescence spectra of TBTPNPs.

The thiophene‐BT‐based D–A polymer (TBT) was successfully prepared (Figure S1, Supporting Information), and the detailed synthesis is summarized in the Supporting Information. Nanoprecipitation induced by DSPE‐mPEG2000 produced water‐soluble TBTPNPs as clear violet solutions. As shown in **Figure** **1**a, the TBTPNPs were spherical with an average size of ≈36 nm (Figure 1b) and showed uniform dispersion without apparent aggregation. The dynamic light scattering (DLS) results showed that the average diameter of the TBTPNPs was 44 nm (Figure 1c), suggesting that the TBTPNPs could generate potential targeting capabilities via enhanced permeability and retention (EPR) effects.^20^ In addition, the as‐prepared TBTPNPs were well dispersed under physiological conditions, such as in phosphate buffered asline (PBS) and Dulbecco's Modifed Eagle Medium (DMEM, high glucose culture medium with serum), without obvious agglomeration and size changes, even after prolonged storage times of up to 60 d at 4 °C (Figure 1c,d). Obtained through ζ‐potential, the surface charge values of the TBTPNPs were –21.2 ± 2 mV in water, –23.7 ± 2 mV in PBS, and –18.9 ± 2 mV in DMEM (Figure 1e). These values may be attributed to the presence of DSPE‐mPEG2000. Moreover, the pH stability of the TBTPNPs was tested using different pH buffer solutions ranging from 2 to 9. The results indicated that no apparent changes occurred in the absorption and fluorescence spectra of the TBTPNPs after incubation for 12 h (Figure S2, Supporting Information). Moreover, no apparent aggregation was observed in the different pH solutions (Figure S3, Supporting Information). These results demonstrated that the TBTPNPs were stable under different physiological conditions.

The UV−vis absorption spectrum of the TBTPNP aqueous solution exhibited a broad absorption band in the range from 400 to 750 nm (Figure 1f). Meanwhile, the NIR emission peak of the TBTPNPs was 690 nm. The optical properties of the TBT polymer in THF were comparable to those of the PT2 polymer in THF. The properties of the TBT polymer were more red shifted than those of the PT2 polymer in THF (Figure S4, Supporting Information). These findings indicated that the introduction of BT units into the skeleton of the thiophene‐based polymer could form D–A structures and lead to red shifted absorption and fluorescence. As measured through an integrating sphere, the absolute fluorescence quantum yield of the TBTPNPs was approximately 0.015 in water. The as‐prepared TBTPNPs in water (xenon lamp, 100 mW cm^−2^ or 635 nm laser, 1 W cm^−2^) and HeLa cells (640 nm laser, laser intensity of 60%) were continuously irradiated under different conditions, and the results showed that the TBTPNPs had high photostability (Figures S5 and S6, Supporting Information).

### 2.2 Light Triggered Generation of ^1^O_2_ and Heat

**Figure 2:**
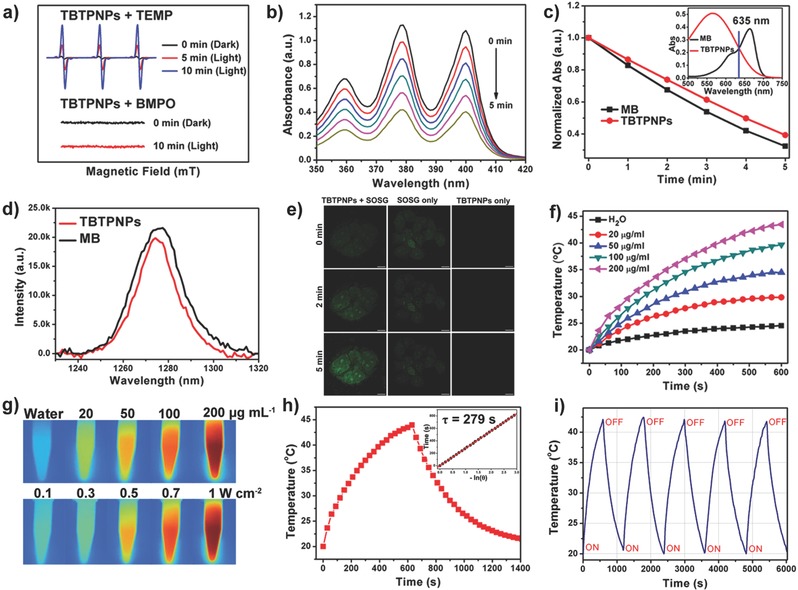
a) Time‐resolved ESR signals of spin traps reacting with ^1^O_2_ (up) and other ROS (down) obtained upon 635 nm laser irradiation of TBTPNPs for 10 min in the presence of TEMP or BMPO. b) Photodegradation of ADPA with TBTPNPs under 635 nm laser irradiation. c) Decay curves of ADPA absorption at 378 nm as a function of time in the presence of TBTPNPs and MB under 635 nm laser irradiation. Insert shows that the absorbance of TBTPNPs and MB are equal at 635 nm. d) ^1^O_2_ luminescence at ≈1,270 nm induced by the TBTPNPs and MB in EtOH under 635 nm laser excitation. e) Evaluation of ^1^O_2_ generation in vitro under 635 nm excitation. Scale bar: 10 µm. f) The concentration and irradiation time‐dependent temperature elevation of TBTPNPs. g) IR images of TBTPNPs with different concentrations under 635 nm laser (1 W cm^−2^) irradiation for 10 min (up) and TBTPNPs (200 µg mL^−1^) with different power densities under 635 nm laser irradiation for 10 min (down). h) Photothermal effect of TBTPNPs (200 µg mL^−1^) exposed to 635 nm laser irradiation (1 W cm^−2^) for 10 min. Insert shows the time constant for heat transfer of the system, which was determined to be τ = 279 s. i) Temperature variations in the TBTPNP solution under 635 nm laser irradiation at the power density of 1 W cm^−2^ for five cycles.

Electron spin resonance (ESR) was initially performed to investigate the ROS production of the TBTPNPs. For this method, 2,2,6,6‐tetramethylpiperidine (TEMP) and 5‐*tert*butoxycarbonyl‐5‐methyl‐1‐pyrroline‐*N*‐oxide (BMPO) were used as ^1^O_2_ and O^2−^· (or OH·) trappers, respectively, under 635 nm laser irradiation. As shown in **Figure** **2**a, a characteristic ^1^O_2_‐induced signal was observed, and the signals of ^1^O_2_ increased with the prolonged irradiation time. In contrast, no other ROS signal (O^2−^· or OH·) was detected (Figure 2a). Subsequently, 9,10‐anthracendipropionic acid (ADPA) was used as a capture probe to evaluate the efficiency of the ^1^O_2_ generation, and methylene blue (MB) was used as the standard photosensitizer (^1^O_2_ quantum yield *Φ*_MB_ = 52% in water).^21^ As shown in Figure 2b and Figure S7 (Supporting Information), the absorption intensity of ADPA at 378 nm gradually decreased as the irradiation time increased in the presence of the TBTPNPs and MB, suggesting ADPA decomposition. According to the decay curves of the ADPA absorption (Figure 2c) and previous reports,[[qv: 21b]] the ^1^O_2_ quantum yield of the TBTPNPs was ≈40%. Sodium azide (NaN_3_), a ^1^O_2_ quencher, was then added into the TBTPNP solutions to demonstrate that the decomposing of ADPA was induced by the generated ^1^O_2_. As shown in Figure S8 (Supporting Information), the decrease in the ADPA absorbance in the present of NaN_3_ was negligible during light irradiation, demonstrating the ^1^O_2_ production of the TBTPNPs. To further exclude the possible direct reaction between ADPA and the TBTPNPs, we subjected the TBTPNPs to a continuous O_2_ and N_2_ purge for 45 min, respectively (Figure S8, Supporting Information). The absorbance of ADPA dramatically decreased in the O_2_‐saturated solution, while it remained nearly unchanged in the N_2_‐saturated solution, indicating that the ^1^O_2_ generation of the TBTPNPs was responsible for the ADPA degradation. The NIR ^1^O_2_ emission around ≈1270 nm was also measured to determine the ^1^O_2_ generation of the TBTPNPs in EtOH.^22^ As shown in Figure 2d, the ^1^O_2_ quantum yield of the TBTPNPs was approximately 43% when MB (*Φ*_MB_ = 49% in ethanol) was used as ref. 21. Meanwhile, ^1^O_2_ sensor green (SOSG), which can selectively react with ^1^O_2_ and form a green fluorescent endoperoxide product, was used to stain fixed cancer cells in order to investigate the ^1^O_2_ generation of the TBTPNPs in vitro. As shown in Figure 2e, the SOSG fluorescence intensity in the fixed HeLa cells treated with TBTPNPs increased considerably during prolonged irradiation times. In contrast, no fluorescence was detected in groups with only SOSG or the TBTPNPs, indicating the efficient ^1^O_2_ generation by the TBTPNPs under irradiation.

Considering the low fluorescence quantum yield of the TBTPNPs, we anticipated that the energy of the TBTPNPs in exited state could be converted to heat via a nonradiative channel.[[qv: 14b]] Thus, various concentrations of TBTPNPs dispersed in water (20, 50, 100, and 200 µg mL^−1^) were exposed to a 635 nm laser at a power density of 1 W cm^−2^ to investigate the photothermal properties of the TBTPNPs. Pure water was used as negative control. The temperatures of all TBTPNP solutions increased with prolonged irradiation times (Figure 2f). After irradiation for 10 min, the temperature of the TBTPNP solutions increased from 9.8 °C (20 µg mL^−1^) to 23.5 °C (200 µg mL^−1^). In contrast, the temperature of pure water increased by only 4.5 °C (Figure 2f,g). Furthermore, 200 µg mL^−1^ aqueous solutions of TBTPNPs were exposed to 635 nm laser at various power densities ranging from 0.1 to 1 W cm^−2^ for 10 min (Figure 2g and Figure S9, Supporting Information). The results indicated that temperature increased rapidly with the increasing laser power. It has been reported that cancer cells can be destroyed by exposure to 42–45 °C for several hours, and this process can be achieved in a few minutes when the temperature exceeds 50 °C (high‐temperature hyperthermia).[[qv: 21b]] Thus, under 0.1 W cm^−2^ laser illumination, the cancer cells hardly reached 42 °C even at 200 µg mL^−1^ TBTPNP concentration. In other words, the photothermal effect can be neglected at such a low laser power level for 10 min irradiation. In contrast, at the same conditions, the cells stained with TBTPNPs at the power density of 1 W cm^−2^ were killed within 10 min because of the high‐temperature hyperthermia effect. We also investigated the ^1^O_2_ generation by TBTPNP (200 µg mL^−1^) sunder 635 nm laser at power density of 1 W cm^−2^. As shown in Figure S10 (Supporting Information), ^1^O_2_ can be formed efficiently, indicating that PDT and PTT effect can be simultaneously achieved under this condition. To calculate the photothermal conversion efficiency of the TBTPNPs, η, the temperature change of the solution (200 µg mL^−1^, 1.0 mL) was recorded as a function of time under continuous irradiation using a 635 nm laser (1 W cm^−2^) until the solution reached a steady‐state temperature (Figure 2h). According to the obtained data and previous reports,^23^ η of the TBTPNPs was calculated as 37.1%. We then evaluated the photothermal stability of the TBTPNPs by monitoring the temperature variations in the TBTPNP solutions under 635 nm laser irradiation for 10 min, and then the solution was cooled to room temperature after the laser was turned off for five cycles. As shown in Figure 2i, even after the fifth cycle, a temperature increase of ≈25 °C was still achieved in 10 min, demonstrating the high photothermal stability and reproducibility of the TBTPNPs. All these results suggested that the obtained TBTPNPs presented PDT and PTT effects under 635 nm laser irradiation, with ≈40% ^1^O_2_ quantum yield, and ≈37.1% photothermal conversion efficiency in water.

### 2.3 In Vitro Imaging and PDT/PTT

**Figure 3:**
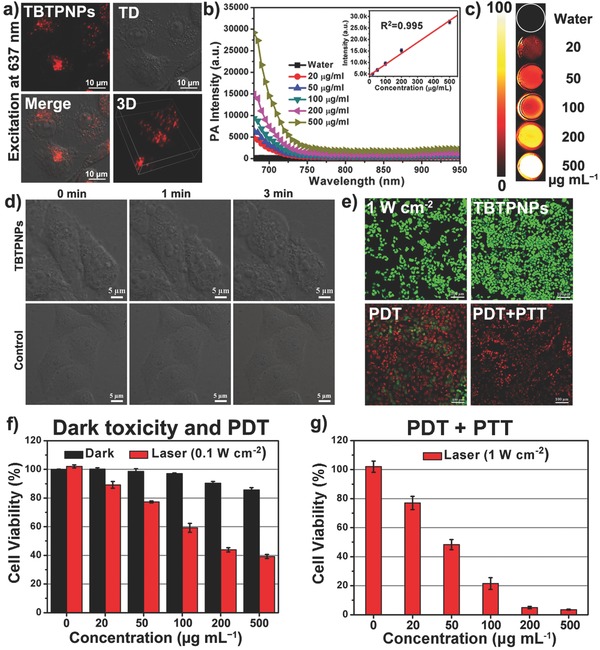
a) Confocal microscopy image of the TBTPNPs in HeLa cells with bright field and 3D imaging excitation at 640 nm. b) PA signals and c) PA imaging of TBTPNPs with different concentrations. Inset in Figure b shows the linear relationship between PA signal intensities and TBTPNPs with different concentrations. d) Time‐dependent confocal bright field images of HeLa cells incubated with and without TBTPNPs after irradiation with 635 nm laser. Scale bar: 20 µm. e) Fluorescence images of calcein AM‐ and PI‐stained HeLa cancer cells with various media: laser only (1 W cm^−2^), TBTPNPs only (200 µg mL^−1^), 200 µg mL^−1^ of TBTPNPs + laser (635 nm, 0.1 W cm^−2^, PDT), and 200 µg mL^−1^ of TBTPNPs + laser (635 nm, 1 W cm^−2^, PDT + PTT). Scale bar = 100 µm. Relative viability of HeLa cancer cells incubated with various concentrations of TBTPNPs under dark or irradiation by 635 nm laser at power densities of f) 0.1 W cm^−2^ (PDT) and g) 1 W cm^−2^ (PDT + PTT) for 10 min.

Owing to their excellent optical properties, the NIR‐emitting TBTPNPs were employed for cell imaging to demonstrate their potential in biological labeling applications by using a confocal laser scanning microscope (CLSM) at excitation wavelength of 640 nm. As shown in **Figure** **3**a, strong NIR fluorescence was observed, particularly in the cytoplasms of the HeLa cells stained with TBTPNPs for 4 h, indicating the efficient uptake by live cells. Z‐stack imaging further demonstrated that the TBTPNPs were localized in the cytoplasm instead of being bound or adsorbed on the cell surface. Given the broad absorption up to the NIR region, the applicability of the TBTPNPs for PA imaging was investigated in an agarose gel phantom (Figure 3b). Figure 3c displays the PA images of the TBTPNPs at concentrations between 0 and 500 µg mL^−1^. The intensities of the PA signals increased with increasing the TBTPNP concentration, as shown in Figure 3b, and a linear relationship between the PA signal intensity and TBTPNP concentration was observed. These data demonstrated that the TBTPNPs can be used as FL and PA imaging agents.

The phototherapy properties of the TBTPNPs against cancer cells were subsequently investigated by monitoring the variations in the morphologies of the HeLa cells in the presence of the TBTPNPs. As shown in Figure 3d, substantial cell morphology changes, including cell shrinkage and formation of numerous blebs, were observed using CLSM upon laser stimulation, indicating cell apoptosis. In contrast, the cells that did not undergo TBTPNP incubation remained unchanged, indicating that the TBTPNPs efficiently generated ^1^O_2_ when subjected to laser irradiation. Calcein AM (green) and PI (red) co‐staining assays, which can distinguish live from dead cells, were further performed to evaluate the photoinduced cell death effect of the TBTPNPs. The controls included samples that underwent laser irradiation (1 W cm^−2^) without TPTPNPs and those with TBTPNPs (200 µg mL^−1^) incubation alone. As shown in Figure 3e, strong green fluorescence signals were present, indicating the safety of the laser beam and the negligible cytotoxicity of the TBTPNPs. However, in the PDT group (0.1 W cm^−2^, 10 min), some parts of the cells were destroyed due to the ease with which ^1^O_2_ was produced and the inability to generate heat under such low laser power doses. When the laser power density was increased to 1 W cm^−2^, all the cells were completely killed, as indicated by the intense homogeneous red fluorescence. This result was ascribed to the fact that both ^1^O_2_ and heat were produced by TBTPNPs at this condition, suggesting that PDT and PTT effects can be achieved and thus can be used to kill cells completely just by using a single laser. The cytotoxicity and light‐triggered synergistic PDT/PTT efficacy of the TBTPNPs on the viabilities of the HeLa cells were further investigated using standard 3‐(4,5‐dimethylthiazol‐2‐yl)‐ 2,5‐diphenyl‐2H‐tetrazolium hydrobromide (MTT) assays. As shown in Figure 3f, without irradiation, cell viabilities of over 85% at 500 µg mL^−1^ TBTPNP concentration were observed, demonstrating the good biocompatibility of the TBTPNPs. However, in the PDT group (0.1 W cm^−2^), the cell viability gradually decreased with the increasing TBTPNP concentration, and ≈60% cell mortality rate was achieved at 500 µg mL^−1^. In the PDT + PTT group (1 W cm^−2^), more than 95% cell mortality rate was observed at TBTPNP concentrations of 200 and 500 µg mL^−1^, thereby verifying that the synergistic PDT and PTT effects could efficiently kill cancer cells (Figure 3g). These results were in good agreement with the findings from the calcein AM and PI costaining experiments.

### 2.4 In Vivo Imaging

**Figure 4:**
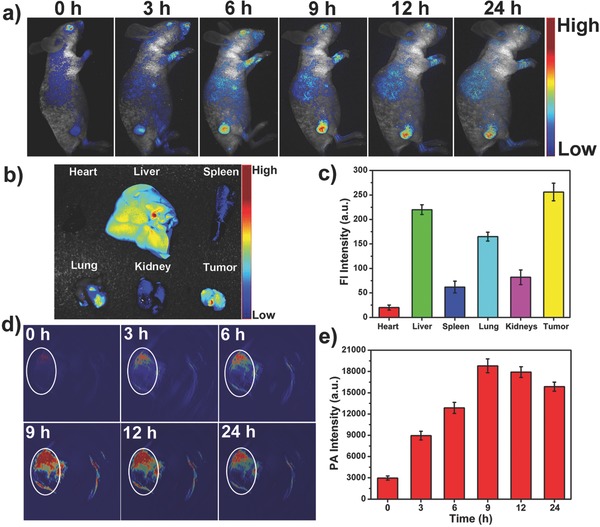
a) Real‐time in vivo FL images after i.v. injection of TBTPNPs in nude mice at different time points. b) Ex vivo images of mice tissues (from top to bottom: heart, liver, spleen, lung, kidneys, and tumor). c) Average FL intensities of mouse tissues at 24 h postinjection (*n* = 5). d) PA images of tumors in mice after i.v. injection of TBTPNPs at different time points. e) Intensities of the average PA signal of the ROI at different time points after i.v. injection.

To evaluate the capability of the TBTPNPs as imaging agents for in vivo NIR FL and PA imaging, a PBS solution containing TBTPNPs (100 µL, 500 µg mL^−1^) was injected into nude mice with HeLa tumors via the tail vein. Initially, the Maestro 2 multispectral small‐animal imaging system was used to record the FL images of the mice. **Figure** **4**a shows the time‐dependent excretion profiles and tumor accumulation of the TBTPNPs in the mice. These data showed that the TBTPNPs accumulated in the tumor area after injection through the EPR effects. The TBTPNP accumulation in the tumors reached its maximum at 9 h postinjection (Figure S10, Supporting Information), and FL was more evident in the tumor area than it was in other tissues. The mice were sacrificed after 24 h postinjection to harvest the main organs (i.e., heart, liver, spleen, lung, and kidneys) and tumors. The organs were then imaged to accurately assess the FL intensities within the tissues (Figure 4b). The results showed that TBTPNPs mainly accumulated in the tumor, kidney, liver, and lung tissues. Meanwhile, negligible signals were detected in the spleen and heart tissues (Figure 4c). We then quantified the dynamic accumulation of the TBTPNPs in the tumor site as a region of interest (ROI) by identifying the time‐dependent FL intensity. As shown in Figure S11 (Supporting Information), the average fluorescence intensity of the accumulated TBTPNPs in the tumor areas reached a plateau at 9 h postinjection and then steadily decreased over time. Subsequently, we examined the in vivo PA imaging performance of the TBTPNPs. As shown in Figure 4d, the tumor site lightened after intravenous (i.v.) injection of the TBTPNPs. Strong PA signals were observed over time and found to be dispersed throughout the whole tumor, in which the average signal exhibited a persistent time‐dependent increase and reached a maximum value at approximately 9 h postinjection (Figure 4e). Therefore, the TBTPNPs can be used as versatile imaging agents for synchronous in vivo FL and PA imaging through i.v. administration.

### 2.5 In Vivo Photothermal Imaging and PDT/PTT

**Figure 5:**
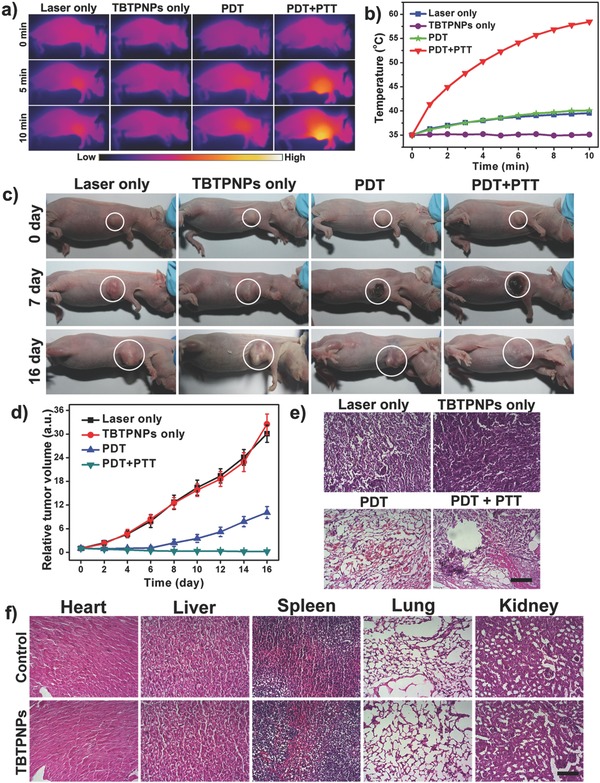
a) IR thermal images of HeLa tumor‐bearing mice in four groups taken at different time intervals. b) Heating curve of the four groups in Figure a. c) Representative photographs of HeLa tumor‐bearing mice after different treatments. d) Time‐dependent tumor growth curves (*n* = 5) observed after different treatments (*n* = 5, *P* < 0.05 for each group) in Figure c. e) H&E staining of tumor sections gathered from various treatment groups of mice on day 2. Scale bar = 100 µm. f) H&E‐stained slices of the heart, liver, spleen, lung, and kidney in mice after PDT + PTT. Scale bar: 100 µm.

To investigate the in vivo therapeutic effect of the TBTPNPs, as a proof of principle experiment, four groups (5 mice per group) of HeLa tumor‐bearing nude mice with tumor sizes of ≈25 mm^3^ were selected as animal models. In the control groups, the mice were only injected with TBTPNPs (TBTPNPs only) or were injected with the same volume of PBS and then subjected to irradiation with a 635 nm laser at laser power density of 1 W cm^−2^ (laser only). For comparison, the mice injected with TBTPNPs and exposed to a 635 nm laser at power densities of 0.1 and 1 W cm^−2^ were selected as the PDT group and PDT + PTT group, respectively. The time evolution of the local tumor temperature during irradiation for 10 min is represented in **Figure** **5**a,b. In the PDT + PTT group, the temperature of the tumor increased significantly from 35 to 58 °C within 10 min. In contrast, the temperatures of the tumors that were only subjected to the laser (1 W cm^−2^) and the PDT groups slightly increased by ≈6 ≈C. We then measured the tumor sizes of the mice from all groups every 2 d after treatment. As shown in Figure 5c, the tumors in the control groups grew significantly during the study period, indicating that laser irradiation or TBTPNP injection alone could not inhibit tumor growth. In contrast, the tumors in the PDT and PDT + PTT groups began to scab and shrink gradually after treatment. Unfortunately, in the PDT group, the tumor began to regrow after 6 d, suggesting that the PDT effect of the TBTPNPs alone was insufficient to completely kill the tumors. However, in the PDT + PTT group, the tumors were completely destroyed, and no tumor relapse was observed over the course of 16 d, revealing a high‐efficiency therapeutic outcome under this condition (Figure 5d). Tumors were also collected for hematoxylin and eosin (H&E) staining on second day posttreatments (Figure 5e). It was clearly observed that all cancer cells were damaged in the PDT + PTT group. To evaluate the TBTPNP toxicity, we recorded the weights of the mice and analyzed the tissue slices. As shown in Figures S12 and S13 (Supporting Information), the mice in the PDT/PTT group did not show abnormal body weight changes and survived over 40 d without a single case of animal death. Moreover, the major mice organs were sliced and stained using H&E for histological analysis. The PDT and the PDT + PTT groups had no substantial damage or inflammation, indicating that the TBTPNPs were not toxic to mice at our tested conditions (Figure 5f).

## 3 Conclusion

In summary, we prepared a novel single nanoplatform for the first time as “all‐five‐in‐one” phototheranostic agent using a facile, one‐step nanoprecipitation method. The developed TBTPNPs exhibited broad absorption that ranged from 400 to 750 nm and an NIR emission peak at ≈690 nm. In addition to possessing the previously reported merits of PNPs, such as high water solubility, excellent light resistance, good pH stability, and negligible toxicity, the prepared TBTPNPs simultaneously possessed PDT and PTT effects under single‐laser irradiation (635 nm), with ≈40% ^1^O_2_ quantum yield and ≈37.1% photothermal conversion efficiency. Furthermore, aside from the two phototherapeutic modalities, FL, PA signal, and thermal imaging using the TBTPNPs were achieved simultaneously. The results of the in vitro and in vivo studies indicated that the as‐prepared TBTPNPs can be used as single versatile phototheranostic agents for trimodal and imaging‐guided simultaneous PDT/PTT. Achieving a better understanding of the in vivo behaviors of the prepared TBTPNPs and their quantitative pharmacokinetics and long‐term toxicity is of extreme importance and requires further exploration. The developed “all‐five‐in‐one” phototheranostic agents in this work may fulfill the “one‐fits‐all” requirement for future phototheranostic applications and thus may provide fundamental insights into the development of PNP‐based nanoagents for cancer therapy.

## 4 Experimental Section

*Materials*: 1,2‐Distearoyl‐sn‐glycero‐3‐phosphoethanolamine‐N‐[methoxy(polyethylene glycol)‐4 2000] was purchased from Shanghai Yanyi Co. SOSG and ADPA were purchased from Sigma‐Aldrich. All other chemicals were obtained from J&K and Beijing Chemical Co. unless otherwise stated Experimental Details.

*Characterization*: Transmission electron microscope (TEM) images were taken on a JEOL JEM‐2100F TEMat an acceleration voltage of 150 kV. UV–Vis and fluorescence spectra were obtained using Hitachi U‐3010 and F‐4500 spectrophotometers, respectively. Zeta potentials were recorded on Zetasize 3000 HS (Malvern, UK). ROS was detected using electron spin resonance technique (ESP300E, Bruker). The fluorescence spectrum of singlet oxygen was detected in HITACHI FL 900 fluorescence spectrophotometer with a NIR detector (λ_ex_ = 635 nm). The fluorescence images of the HeLa cells were acquired with a Nikon A1R confocal and multiphoton system using 640 nm laser and an oil immersion 60× objective lens.

*Preparation of TBTPNPs*: A solution of polymer TBT in tetrahydrofuran (THF) (1 mg mL^–1^, 1 mL) and a solution of DSPE‐mPEG2000 in water (5 mg mL^–1^, 1 mL) were rapidly injected into distilled‐deionized water (9 mL) under continuous sonication for 3 min. THF was subsequently removed and the TBTPNPs were further purified through dialysis and filtered through a 0.22 µm filter membrane.

*Photostability of TBTPNPs In Vitro*: HeLa cells (donated by the Center of Cells, Peking Union Medical College) were cultured in culture media (DMEM/F12 supplemented with 10% fetal bovine serum, 50 unit mL^−1^ of penicillin, and 50 mg mL^−1^ of streptomycin) at 37 °C in a humidified incubator containing 5% CO_2_. The cells were then stained with TBTPNPs in culture media for 6 or 1 h at 37 °C. The cells were washed with PBS and then fixed with ice methanol for 1 min, carefully washed with PBS again for three times, and then irradiated and imaged using NIS element analysis with a cooled CCD camera.

^1^ *O*_2_ *Quantum Yield Measurements via Chemical Method*: ADPA was used as the ^1^O_2_‐trapping agent, and MB was used as the standard photosensitizer. In the experiments, 60 µL of ADPA solution (1 mg mL^−1^) was added to 1.5 mL of TBTPNPs solution, and 635 nm laser was employed as the irradiation source. To eliminate the inner‐filter effect, the absorption maxima of MB and the TBTPNPs were adjusted to ≈0.2 OD. The absorption of ADPA at 378 nm was recorded at various irradiation times to obtain the decay rate of the photosensitizing process. The ^1^O_2_ quantum yield of the TBTPNPs in water was calculated using the following formula

(1) $$\Phi_{{\mathrm{TBTPNP}}_{S}}=\;\;\Phi_{\mathrm{MB}}\frac{t_{\mathrm{MB}}}{t_{{\mathrm{TBTPNP}}_{S}}}$$

where *t*_MB_ is the time for the decrease in absorption of ADPA in the presence of MB free in water adjusted to a first‐order exponetial decay, *t*_TBTPNPs_ is the time for the decrease in absorption of ADPA in the presence of TBTPNPs in water adjusted to a first‐order exponetial decay, and *Φ*_MB_ is the singlet oxygen quantum yield of MB free in water given as 0.52.

^1^ *O*_2_ *Quantum Yield Measurements by Detecting* ^1^ *O*_2_ *Emission*: The ^1^O_2_ emission signals of the TBTPNPs were detected in a HITACHI FL 900 fluorescence spectrophotometer with a 635‐nm excitation laser and a NIR detector. Considering the short PL lifetime of ^1^O_2_ in water, the MB and TBTPNPs solution were prepared in EtOH solution. The absorptions of MB and TBTPNPs at 635 nm were adjusted to ≈0.2 OD. The ^1^O_2_ quantum yield of the PNPs could be obtained using the following

(2) $$\Phi_{{\mathrm{TBTPNP}}_{S}}=\;\;\frac{I_{\mathrm{TBTPNPS}}}{I_{\mathrm{MB}}}\;\;\Phi_{\mathrm{MB}}$$

where *I*_TBTPNPs_ and *I*_MB_ represent the PL peak areas of ^1^O_2_ produced by the TBTPNPs and MB, respectively, and φ_MB_ = 0.49.

*Measurement of Photothermal Performance and Calculation of the Photothermal Conversion Efficiency*: Various solutions of TBTPNPs (1.0 mL) with different concentrations (0 –200 µg mL^−1^) were introduced in a quartz cuvette and irradiated with a 635 nm laser at a power density of 1 W cm^−2^ for 10 min at room temperature to measure the photothermal conversion performance. The temperature changes of TBTPNPs (200 µg mL^−1^) were also measured under irradiation with the 635 nm laser at different powder density. The temperature was recorded every 1 s with a digital thermometer using a thermocouple probe with an accuracy of 0.1 °C. In order to calculate photothermal conversion efficiency η, which defined as the fraction of the absorbed power that is transformed into heat, the temperature during a cycle of laser‐induced heating and subsequent cooling of an aqueous dispersion of TBTPNPs was measured and then using the equation

(3) $$\eta =\frac{hA\Delta T_{\max}-Q_{\mathrm{DIS}}}{I(1-10^{-A\lambda })}$$

where *h* is the heat transfer coefficient and *A* is the surface area of the container where the solution is placed, while Δ*T*_max_ is the temperature change of the TBTPNPs solution at the maximum steady‐state temperature. *Q*_DIS_ corresponds to the heat dissipated from the light absorbed by the solvent and container, *I* is the laser power, and *A_λ_* is the optical density of the TBTPNPs solution at 635 nm. And the thermal imaging was performed using a Ti400 infrared camera when the tumors were exposed to lasers.

*PA Imaging In Vitro*: The TBTPNPs with different concentrations (0, 20, 50, 100, 200, and 500 µg mL^–1^) were added into the agarose tube (37 °C) with the same concentration of micelles as the control. Then, they were scanned with a PA imaging instrument (mode: iTheraMedical Co. MOST inVision 128; excitation wavelength ranged from 680 to 980 nm with 5 nm interval), and the PA signal was recorded through a mean pixel intensity of the same area in the images.

*Cell Imaging and PDT/PTT In Vitro*: HeLa cancer cells were seeded in 35 mm cell culture dishes and were incubated with TBTPNPs (1 mg mL^−1^, 50 µL). After 4 h, cells were washed with PBS twice to remove nonspecifically bound TBTPNPs. Images were acquired with a Nikon C1si laser scanning confocal microscope. For PDT/PTT in vitro, HeLa cancer cells were incubated with and without TBTPNPs (1mg mL^−1^ 50 µL) for 6 h and then irradiated by 635 nm laser at different power densities (0.1, and 1 W cm^−2^) for 10 min. After that, the cells were costained with calcein AM and propidium iodide (PI) for 10 min, washed with PBS, and then imaged by a Nikon C1si laser scanning confocal microscope. To determine cell viabilities, MTT assay was carried out under various conditions to further confirm the cytotoxicity and phototherapy efficacy of TBTPNPs. Cells were seeded into 96‐well plates and incubated with different concentrations (0, 20, 50, 100, 200, and 500 µg mL^−1^) of TBTPNPs at 37 °C for 24 h in 5% CO_2_ atmosphere, each concentration of TBTPNPs was carried out with six parallel groups. For in vitro PDT and PDT/PTT, HeLa cancer cells were incubated with TBTPNPs (0, 50, 100, 200, and 500 µg mL^−1^) at 37 °C for 6 h under the same conditions and then irradiated by 635 nm laser (0, 0.1 W cm^−2^ and 1 W cm^−2^) for 10 min. After illumination, the cells were incubated for another 24 h. The cell medium solutions were exchanged for 100 µL of fresh medium, followed by the addition of 20 µL of MTT solution to each well. The culture plates were then incubated at 37 °C in 5% CO_2_ for 4 h. The culture medium was discarded, and 100 µL of dimethyl sulfoxide (DMSO) was added. The absorbance of the untreated cell population under the same experimental conditions was used as the reference point to establish 100% cell viability.

*FL/PA/Thermal Imaging In Vivo*: Animal experiments were approved by the China Committee for Research and Animal Ethics in compliance with the law on experimental animals. A subcutaneous HeLa tumor was established by injecting a suspension of 2 × 10^7^ HeLa cells in PBS (100 µL) into the buttock of each female nude mouse (4 week old, 15−20 g) and was allowed to grow for 8–10 d when the tumor size reached 20–25 mm^3^. The TBTPNPs solution (500 µg mL^−1^, 100 µL) was intravenously injected into tumor‐bearing mice. The fluorescence images of mice were then recorded on a Maestro 2 Multispectral Small‐animal Imaging System. Excitation wavelength was set at 570 nm. Fluorescent scans were performed at various time points (0, 3, 6, 9, 12, and 24 h) postinjection. The tumor‐bearing mice were sacrificed by exsanguinations at 24 h postinjection, and the tumor and major organs were harvested. The in vivo PA imaging was performed on a commercial MSOT InVision128 PA tomography system (iThera Medical, Germany). The system houses 128 unfocused ultrasound transducers (with 5 MHz center frequency and 3 mm diameter) arranged in a hemispherical bowl filled with water and a temperature monitor of the water bath (37 °C). Prior to intravenous administration of the TBTPNPs (500 µg mL^–1^, 100 µL), we first obtained precontrast data with excitation wavelength from 680 to 980 nm. Then, postcontrast data were acquired at various time points (3, 6, 9, 12, and 24 h) after intravenous administration of TBTPNPs under the same scan wavelength ranges. The PA images were reconstructed offline using data acquired from all 128 transducers at each view through a modified back‐projection algorithm. Thermal imaging was recorded by a Ti400 infrared camera when the tumors were exposed to 635 nm laser with a power density of 0.1 or 1 W cm^−2^ for 10 min.

*Phototherapy In Vivo*: When the tumor size reached 20–25 mm^3^, the HeLa tumor mice were randomly divided into four groups (5 mice per group). For the treatment groups, mice were intravenously injected with TBTPNPs solution (500 µg mL^−1^, 100 µL) and were covered by home‐made baffle with hole of 5 × 5 mm^2^, which could cover the normal tissue and expose the tumor tissue under irradiation. Then, mice were irradiated by 635 nm laser (0.1 W cm^−2^, PDT or 1 W cm^−2^, PDT/PTT) for 10 min. The control groups included mice with TBTPNPs injection only and mice with laser irradiation only (1 W cm^−2^ of laser only). Tumor sizes, body weight, and survival rate were measured every other day after treatment. Tumor volume (*V*) was determined by Equation (4)

(4) $$V=\frac{ab^{2}}{2}$$

where *a* and *b* are the length and width of the tumor, respectively. The relative tumor volume was normalized to its initial size before administration and laser irradiation of BTTPNPs.

*Histopathological Examination*: The tissues (heart, liver, spleen, kidneys, and lung) were harvested and fixed in 10% formalin solution. The histopathological tests were performed according to standard laboratory procedures. Tissue samples were numbered and given blind to a pathologist for conventional processing and analysis. The tissue samples were embedded in paraffin blocks, sectioned into 5 µm slices, and mounted onto the glass slides. After H&E staining, the sections were observed, and photos were taken using an optical microscope (20× magnification: liver, heart, spleen, lung, and kidney).

## Conflict of Interest

The authors declare no conflict of interest.

## Supporting information

Supplementary
