# Supplementary material for: Single Near‐Infrared Emissive Polymer Nanoparticles as Versatile Phototheranostics
Source: Adv Sci (Weinh). 2017 Jun 10;4(10):1700085. doi: 10.1002/advs.201700085 (PMC5644228; doi:10.1002/advs.201700085)
Supplement: Supplementary file 1 — Supplementary [file ADVS-4-na-s001.pdf]

Copyright WILEY-VCH Verlag GmbH & Co. KGaA, 69469 Weinheim, Germany, 2017.

ADVANCED SCIENCE
Open Access

Supporting Information

for *Adv. Sci.,* DOI: 10.1002/advs.201700085

Single Near-Infrared Emissive Polymer Nanoparticles as Versatile Phototheranostics

Liang Guo, Guangle Niu, Xiuli Zheng, Jiechao Ge, Weimin Liu, Qingyan Jia, Panpan Zhang, Hongyan Zhang, and Pengfei Wang**

Copyright WILEY-VCH Verlag GmbH & Co. KGaA, 69469 Weinheim, Germany, 2016.

Supporting Information

Single Near-infrared Emissive Polymer Nanoparticles as a Versatile Phototheranostics

Liang Guo,[1,2†] Guangle Niu,[1,2†] Xiuli Zheng,[1,2] Jiechao Ge,[1,2] Weimin Liu,[1,2] Qingyan Jia,[1,2] Panpan Zhang,[1] Hongyan Zhang,[1] and Pengfei Wang[1,2*]*

[†]These authors contributed equally to this work.

1. Synthesis.

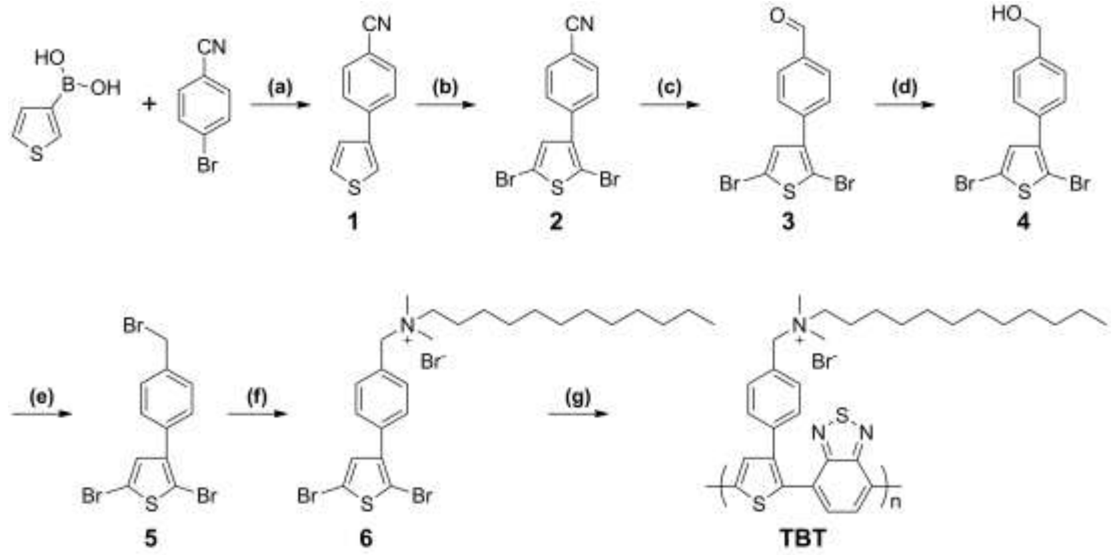

Conditions: (a) Na_2CO_3, $Pd(PPh_3)_4$, EtOH, 90 °C, 8 h; (b) NBS, DMF, rt, 24 h; (c) DIBAL, CH_2Cl_2, reflux, 6 h; (d) $NaBH_4$, THF, rt, 24 h; (e) NBS, PPh_3, THF, rt, 1 h; (f) *N*,*N*-Dimethyldodecylamine, AcOEt, rt, 2 h; (g) 4,7-Bis(4,4,5,5-tetramethyl-1,3,2-dioxaborolan-2-yl)-2,1,3-benzothiadiazole, Na_2CO_3, $Pd(PPh_3)_4$, 96 °C, 72 h.

Figure S1. The synthetic route of polymer TBT.

1.1 Synthesis of compound 1: Deionized water (20 mL) was added with syringes to a mixture of 4-bromobenzonitrile (0.326 g, 1.8 mmol), Na_2CO_3 (0.840 g, 8 mmol), $Pd(PPh_3)_4$ (208 mg, 0.18 mmol), and thiophene-3-boronic acid (0.256 g, 2 mmol) in EtOH (50 mL) under nitrogen. Then the mixture was refluxed at 90 °C for 8 h. After cooling to room temperature, EtOH was removed under reduced pressure. The residue was extracted with CH_2Cl_2 (3 × 20 mL) and the resulting organic layer was collected and dried with anhydrous $MgSO_4$. After removal of the solvent under reduced pressure, the residue was purified by column chromatography (eluent: petroleum ether (PE)/ethyl acetate (EA) = 2:1) to give compound 1 (0.250 g, yield 75 %) as a white solid. ^{1}H NMR (400 MHz, $CDCl_3$): δ 7.69 (s, 4H), 7.58 (s, 1H), 7.47 – 7.39 (m, 2H). ^{13}C NMR (100 MHz, $CDCl_3$): δ 140.38, 140.04, 132.71, 127.17, 126.86, 125.94, 122.61, 118.94, 110.53. TOF-EI calcd for [$C_{11}H_7NS$] m/z 185.0299 (M), found 185.0300.

1.2 Synthesis of compound 2: A solution of *N*-bromosuccinimide (1.24 g, 7 mmol) in 5 mL of anhydrous DMF was added dropwise to the solution of compound 1 (0.555 g, 3 mmol) in 15 mL of anhydrous DMF under N_2 protection. The solution was stirred at room temperature for 24 h and then water was added slowly. The resulting mixture was further stirred for 1h and extracted with diethyl ether. The combined organic extracts were dried over anhydrous $MgSO_4$ and the solvents were removed under reduced pressure. The remaining residue was purified by column chromatography (PE/EA=10/1) to give compound 2 (0.982 g, 96 %) as a white solid. ^{1}H NMR (400 MHz, DMSO-d_6) δ 7.96 (d, J = 7.7 Hz, 2H), 7.77 (d, J = 7.7 Hz, 2H), 7.50 (s, 1H). ^{13}C NMR (100 MHz, DMSO-d_6): δ 140.19, 137.81, 132.52, 132.15, 129.31, 118.53, 111.64, 110.81, 109.18. TOF-EI calcd for [$C_{11}H_5Br_2NS$] m/z 340.8509 (M), found 340.8513.

1.3 Synthesis of compound 3: Diisobutylaluminium hydride dichloromethane solution (1M, 6.0 mL, 6.0 mmol) was added dropwise to the solution of compound 2 (1.95 g, 5.7 mmol) in 60 mL of anhydrous CH_2Cl_2 under N_2 protection. The solution was reflux for 6 h. After cooling to room temperature, methanol was then carefully added until the crude production was precipitated. After addition of water, the reaction crude was treated with concentrated HCl until complete solution and extracted with chloroform. The combined organic extracts were washed with water and dried over anhydrous $MgSO_4$. After removal of the solvent under reduced pressure, the remaining residue was purified by column chromatography (PE/EA=10/1) to afford compound 3 (1.83 g, 93 %) as a white solid. ^{1}H NMR (400 MHz,

$CDCl_3$) δ 10.06 (s, 1H), 7.95 (d, *J* = 8.3 Hz, 2H), 7.68 (d, *J* = 8.2 Hz, 2H), 7.06 (s, 1H). ^{13}C NMR (100 MHz, DMSO-d_6): δ 192.63, 140.75, 138.90, 135.48, 132.23, 129.64, 129.12, 111.55, 108.88. TOF-EI calcd for [$C_{11}H_6Br_2OS$] m/z 343.8506 (M), found 343.8526.

1.4 Synthesis of compound 4: $NaBH_4$ (0.092 g, 2.43 mmol) was added in portions to the solution of compound 3 (0.691 g, 2 mmol) in 30 mL of anhydrous THF under N_2 atmosphere. The mixture was stirred at room temperature for 24 h, and then water was added. The mixture was extracted with diethyl ether and the combined organic extracts were dried over $MgSO_4$. After removal of the solvent under reduced pressure, the residue was purified by column chromatography (CH_2Cl_2) to afford compound 4 (0.625 g, 90 %) as a white solid. ^1H NMR (400 MHz, $CDCl_3$) δ 7.50 (d, *J* = 8.2 Hz, 2H), 7.43 (d, *J* = 8.2 Hz, 2H), 7.01 (s, 1H), 4.75 (s, 2H), 1.65 (s, 1H).^{13}C NMR (100 MHz, DMSO-d_6): δ 142.69, 141.88, 132.32, 131.64, 128.09, 126.54, 110.93, 107.01, 62.55. TOF-EI calcd for [$C_{11}H_8Br_2OS$] m/z 345.8663 (M), found 345.8591.

1.5 Synthesis of compound 5: A solution of NBS (0.585 g, 3.3 mmol) in 10 mL of anhydrous THF was slowly added over 20 min to the solution of compound 4 (0.763 g, 2.2 mmol) and PPh_3 (0.864 g, 3.3 mmol) in 25 mL of anhydrous THF under N_2 atmosphere. After stirring for 1 h at room temperature, the reaction crude was filtered and the filtrate was evaporated under reduced pressure. Then the residue was purified by column chromatography (PE/EA= 15/1) to afford compound 5 as a white solid (0.776 g, 86 %). ^1H NMR (400 MHz, $CDCl_3$) δ 7.50 – 7.43 (m, 4H), 7.01 (s, 1H), 4.53 (s, 2H). ^{13}C NMR (100 MHz, $CDCl_3$): δ 141.33, 137.60, 134.16, 131.55, 129.21, 128.94, 111.48, 108.06, 32.98. TOF-EI calcd for [$C_{11}H_7Br_3S$] m/z 407.7819 (M), found 407.7835.

1.6 Synthesis of compound 6: *N*,*N*-Dimethyldodecylamine (0.554 g, 2.6 mmol) was added to the solution of compound 5 (0.818 g, 2 mmol) in 25 mL AcOEt. After stirring for 2 h at room temperature, white precipitate was formed and then was filtered and washed with plenty of AcOEt. The white precipitate of compound 6 (1.144 g, 92 %) was dried and used in the next step without further purification. ^1H NMR (400 MHz, $CDCl_3$) δ 7.75 (d, *J* = 7.4 Hz, 2H), 7.54 (d, *J* = 7.6 Hz, 2H), 6.96 (s, 1H), 5.24 (s, 2H), 3.63 – 3.50 (m, 2H), 3.33 (s, 6H), 1.81 (s, 2H), 1.41 – 1.15 (m, 18H), 0.87 (t, *J* = 6.2 Hz, 3H).^{13}C NMR (100 MHz, $CDCl_3$): δ 140.74, 138.03, 133.80, 126.91, 126.79, 125.99, 125.84, 121.65, 67.13, 63.74, 49.58, 31.88, 29.56, 29.43,

29.38, 29.30, 29.26, 26.33, 22.94, 22.66, 14.09. HR-ESI calcd for $[C_{25}H_{38}Br_2NS]^+$ m/z 544.10862 ($[M-Br]^+$), found 544.10820.

1.7 Synthesis of polymer TBT polymer: Compound 6 (0.310 g, 0.5 mmol), 4,7-Bis(4,4,5,5-tetramethyl-1,3,2-dioxaborolan-2-yl)-2,1,3-benzothiadiazole (0.194 g 0.5 mmol), $Pd(PPh_3)_4$ (0.023 g, 0.02 mmol), Na_2CO_3 (0.265 g, 2.5 mmol) were added to toluene (10 mL) under N_2 atmosphere. Then the mixture was heated at 96 °C for 72 h. After cooling to room temperature, the mixture was added into MeOH and precipitate was formed. The precipitate was then washed by hot water and hot methanol several times. The final polymer TBT (0.248 g, 84 %) was dried under vacuum for the further experiments. Elem. anal. calcd for $C_{31}H_{42}BrN_3S_2$: C, 61.98; H, 7.05; N, 6.99, found: C, 61.57; H, 7.13; N, 6.72.

2. pH stability of TBTPNPs.

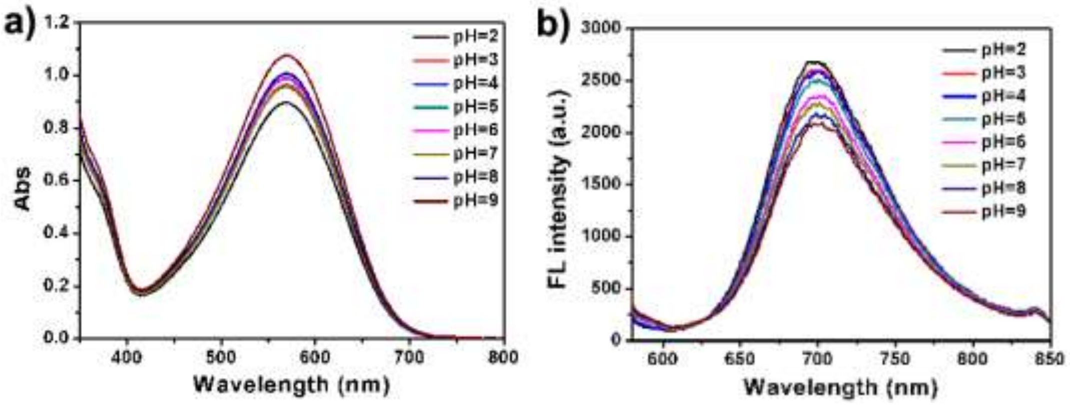

Figure S2. The absorption (a) and fluorescence (b) spectra of TBTPNPs in different pH (from 2 to 9) solutions.

3. DLS of TBTPNPs in different pH solutions.

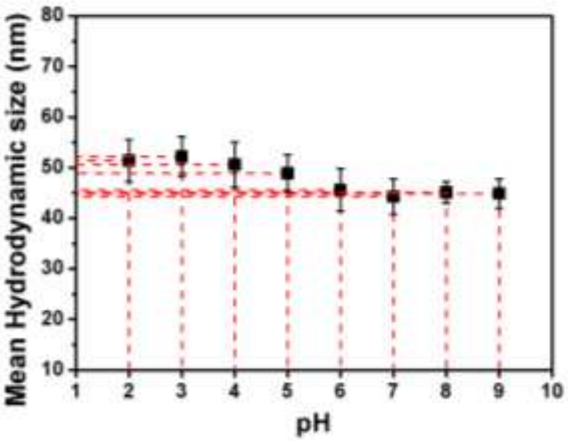

Figure S3. DLS of TBTPNPs in different pH solutions.

4. UV–vis and fluorescence spectra of polymer PT2 and TBT in THF.

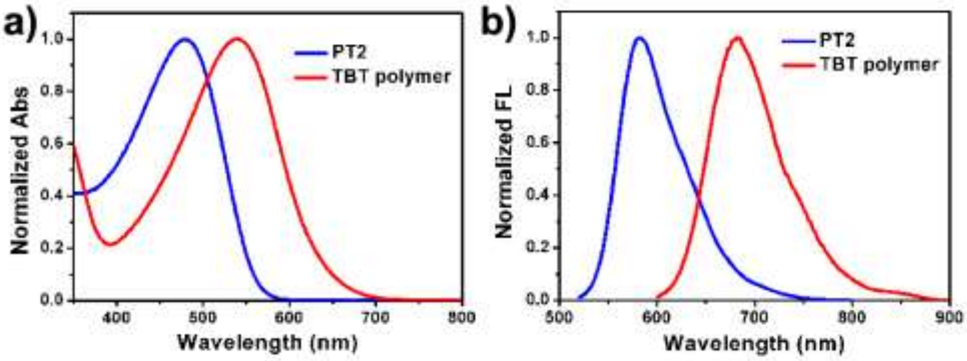

Figure S4. UV–vis (a) and fluorescence (b) spectra of polymer PT2 and TBT in THF.

5. Photostability of TBTPNPs in water.

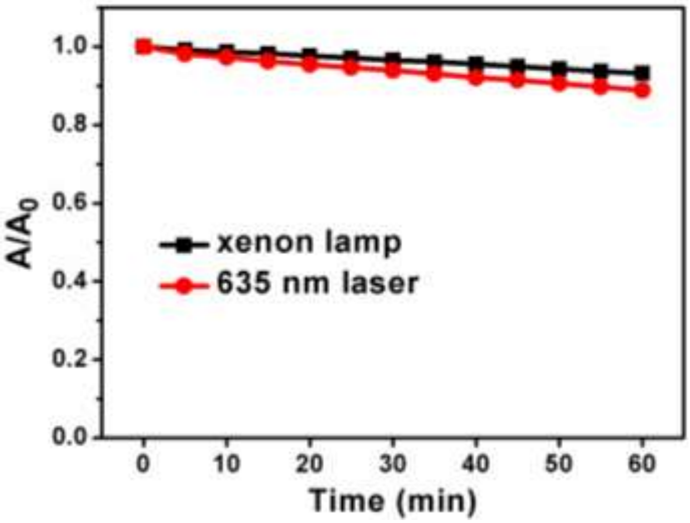

Figure S5. Evolution of the photostability of TBTPNPs in water.

6. Photostability of TBTPNPs *in vitro*.

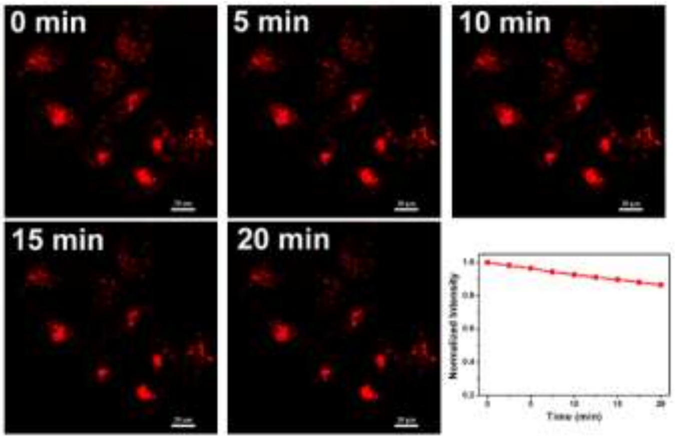

Figure S6. Confocal microscopy images showing time-dependent fluorescence of fixed HeLa cells incubated with TBTPNPs (100 μg mL^{-1} 50 μL). Scale bar = 20 μm.

7. Photodegradation of ADPA with MB.

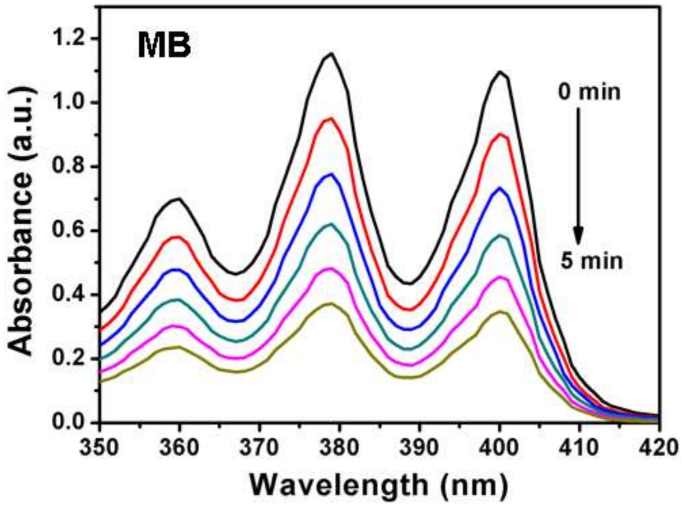

Figure S7. Photodegradation of ADPA with MB.

8. Normalized absorbance of the ADPA in different conditions.

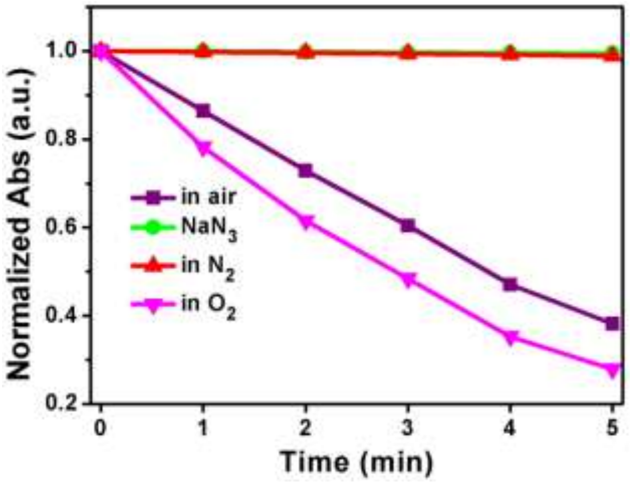

Figure S8. Normalized absorbance of the ADPA in the presence of TBTPNPs in different conditions.

9. Laser power dose-dependent temperature elevation of TBTPNPs (200 μg mL^{-1}) under 635 nm laser irradiation for 10 min.

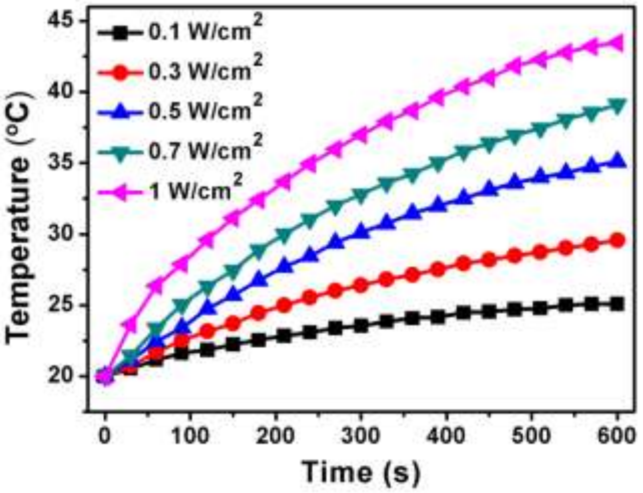

Figure S9. Laser power dose-dependent temperature elevation of TBTPNPs (200 μg mL^{-1}) under 635 nm laser irradiation for 10 min.

10. $^1O_2$ generation under 635 nm laser at power density of 1 W cm^{-2}.

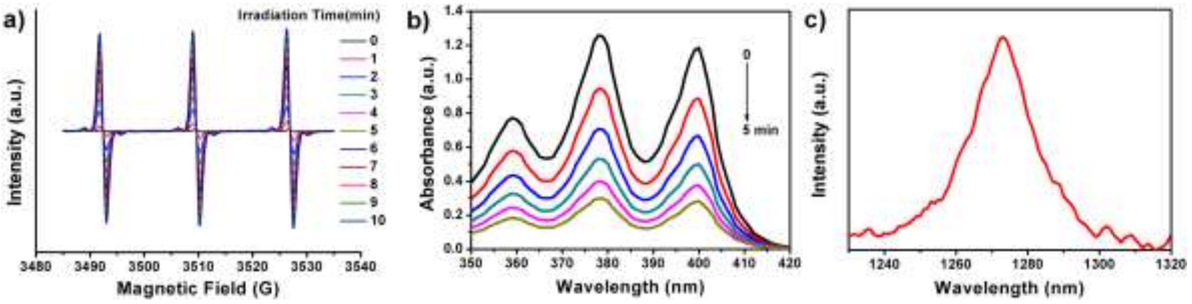

Figure S10. Time-resolved ESR signals of spin traps reacting with $^1O_2$ obtained upon 635 nm laser irradiation of TBTPNPs for 10 min in the presence of TEMP; b) Photodegradation of ADPA with TBTPNPs under 635 nm laser irradiation; c) $^1O_2$ luminescence at ~1,270 nm induced by the TBTPNPs in EtOH under 635 nm laser excitation.

11. Intensities of the mean FL signal of the ROI at different time points (0–24 h) after *i.v.* injection.

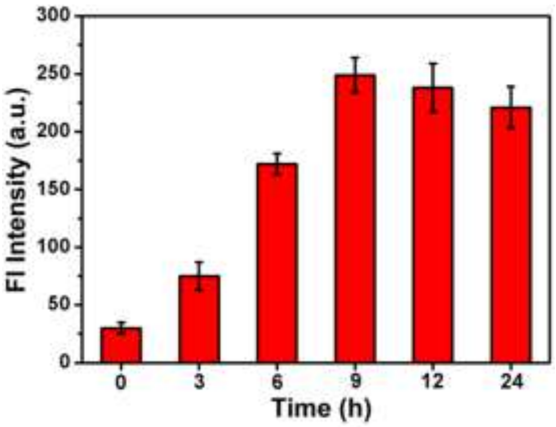

Figure S11. Intensities of the mean FL signal of the ROI at different time points (0–24 h) after *i.v.* injection.

12. Body weight measurements of mice after various treatments.

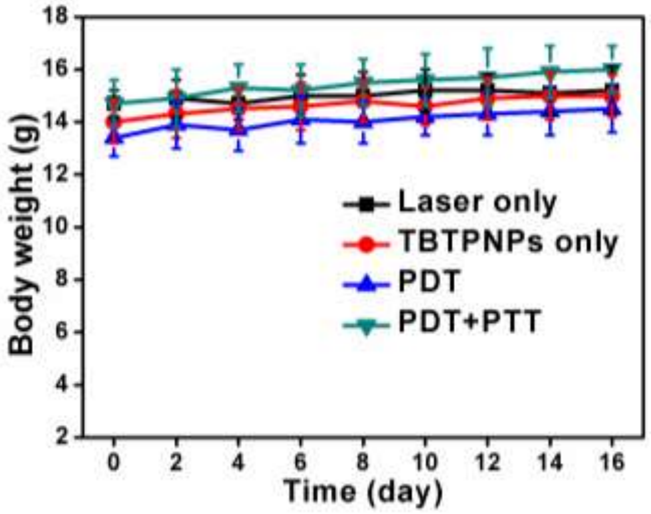

Figure S12. Body weight measurements of mice after various treatments.

13. Survival curves of mice after various treatments.

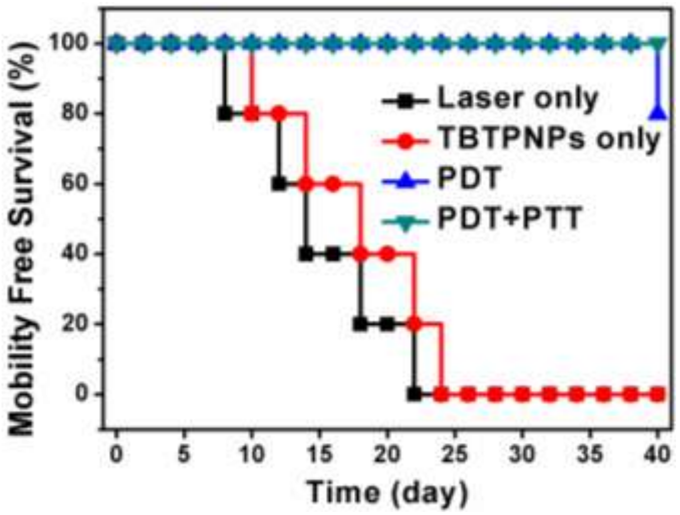

Figure S13. Survival curves of mice after various treatments.

14. NMR spectra

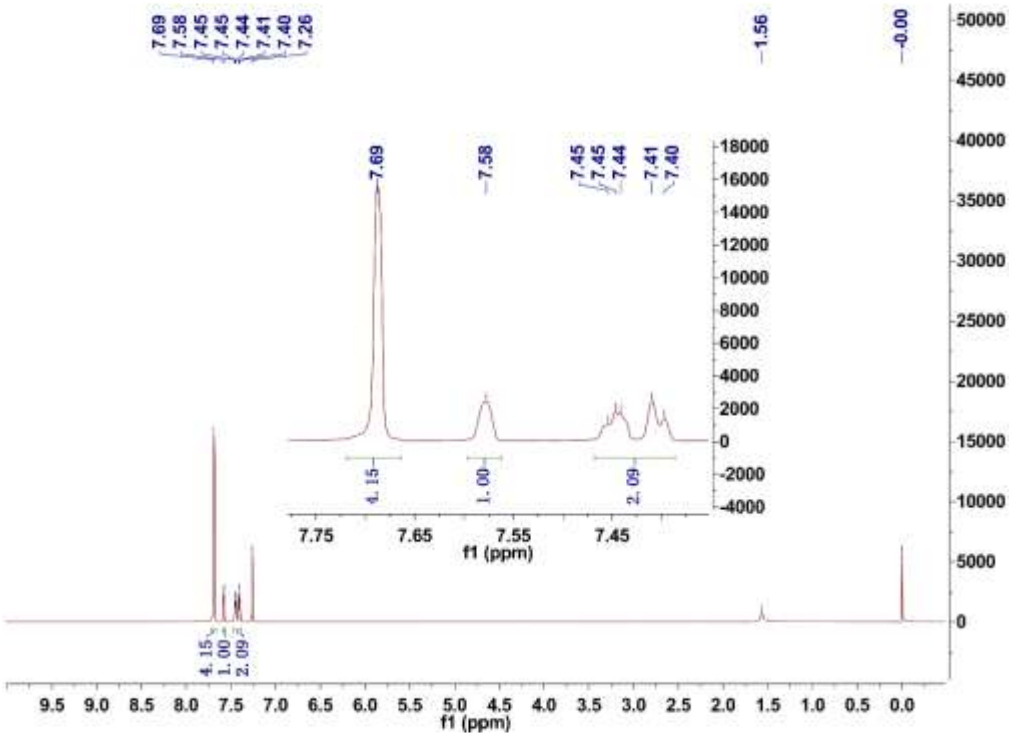

Figure S14. ^{1}H-NMR (400 MHz, $CDCl_3$): Compound 1.

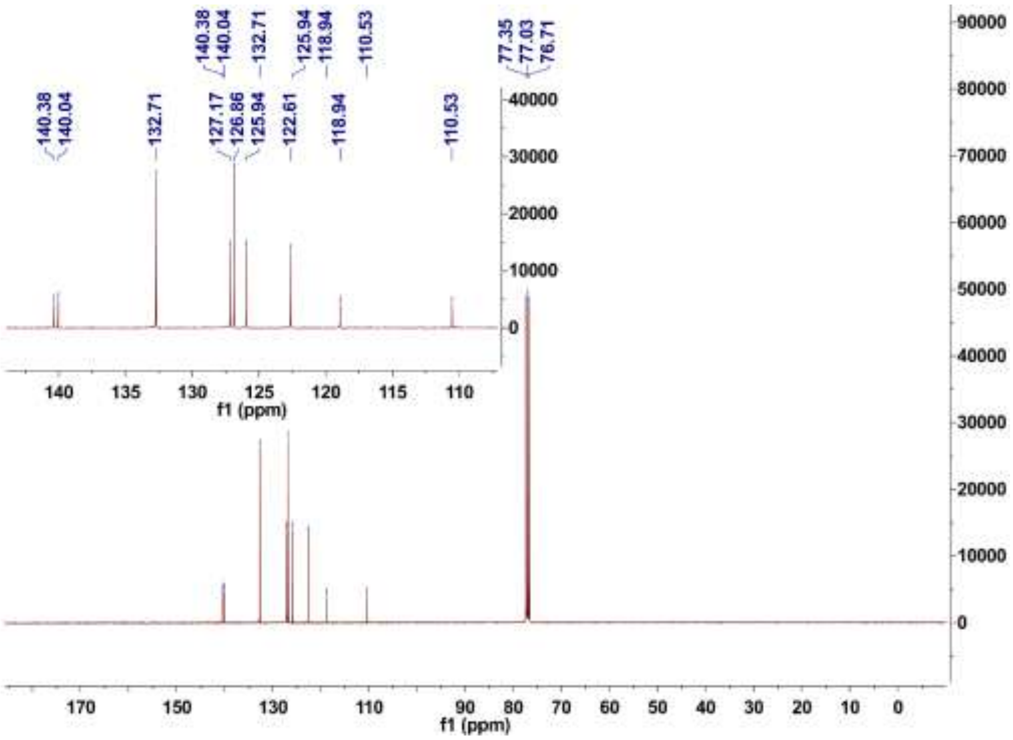

Figure S15. ^{13}C-NMR (100 MHz, $CDCl_3$): Compound 1.

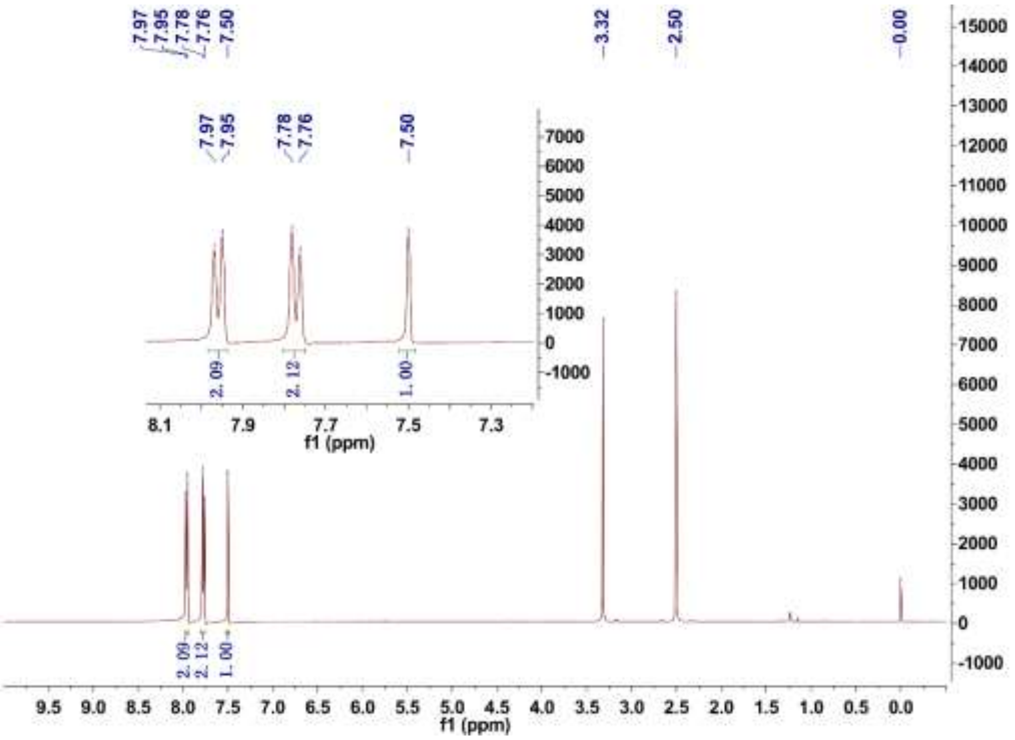

Figure S16. ^{1}H-NMR (400 MHz, DMSO-d_6): Compound 2.

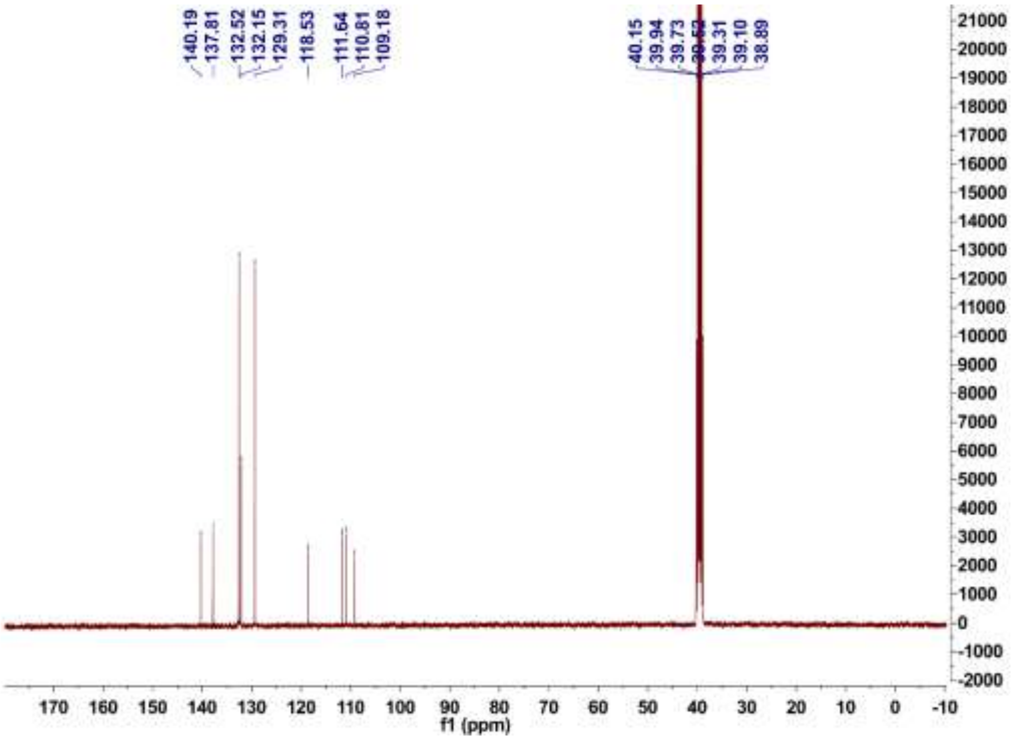

Figure S17. ^{13}C-NMR (100 MHz, DMSO-d_6): Compound 2.

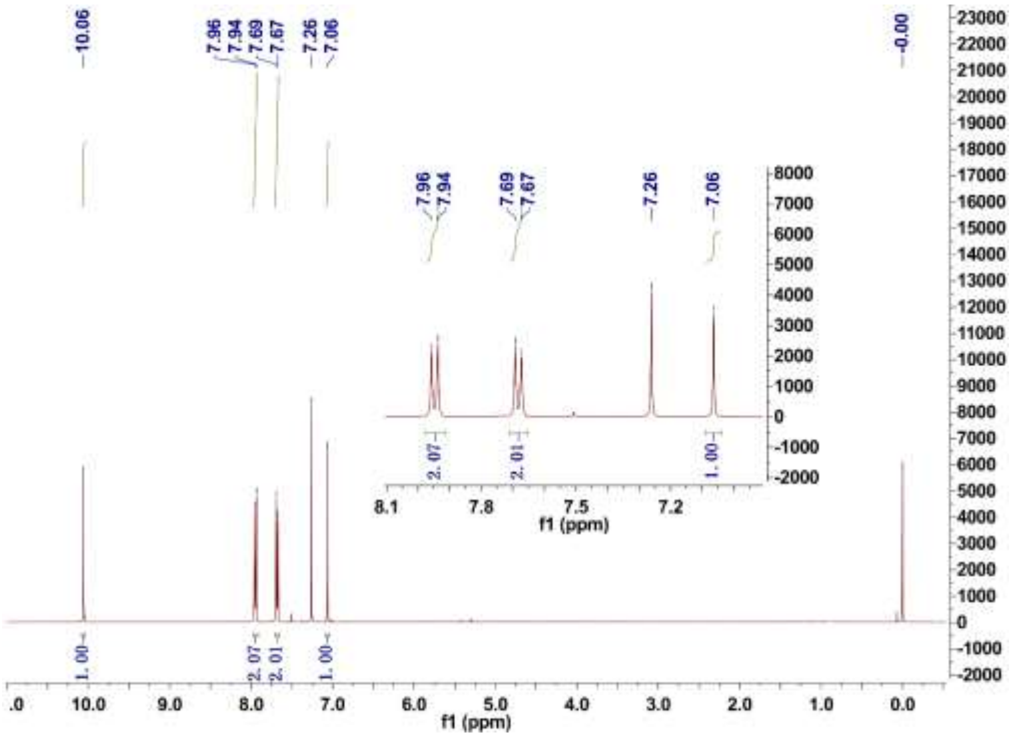

Figure S18. ^{1}H-NMR (400 MHz, $CDCl_3$): Compound 3.

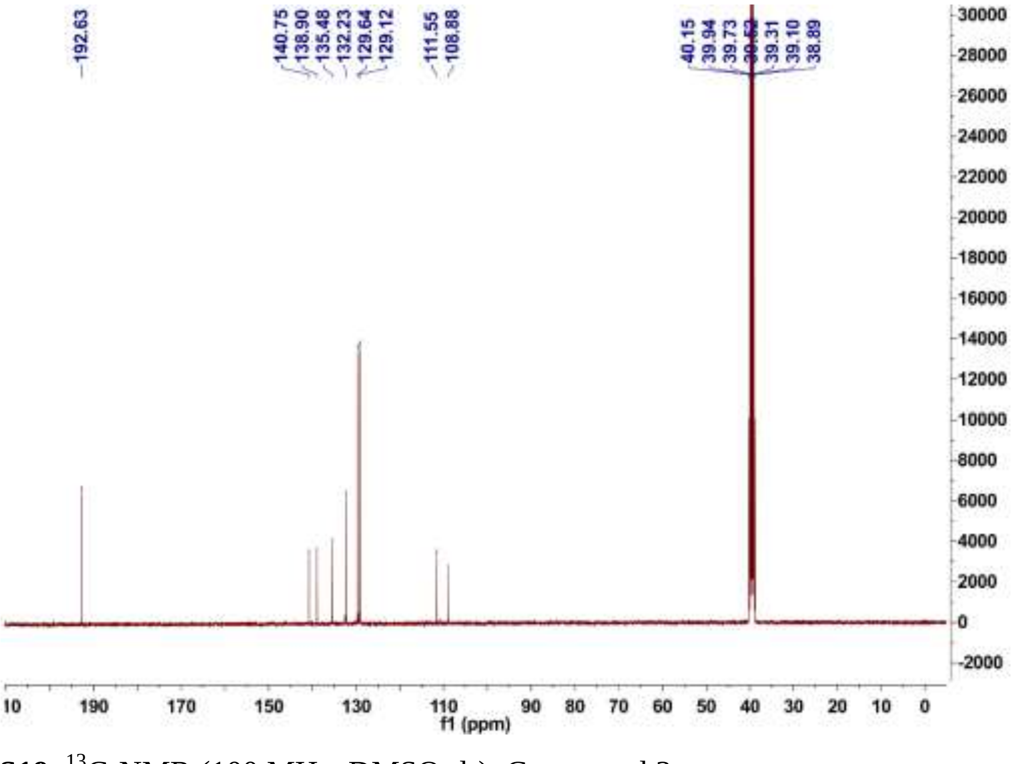

Figure S19. ^{13}C-NMR (100 MHz, DMSO-d_6): Compound 3.

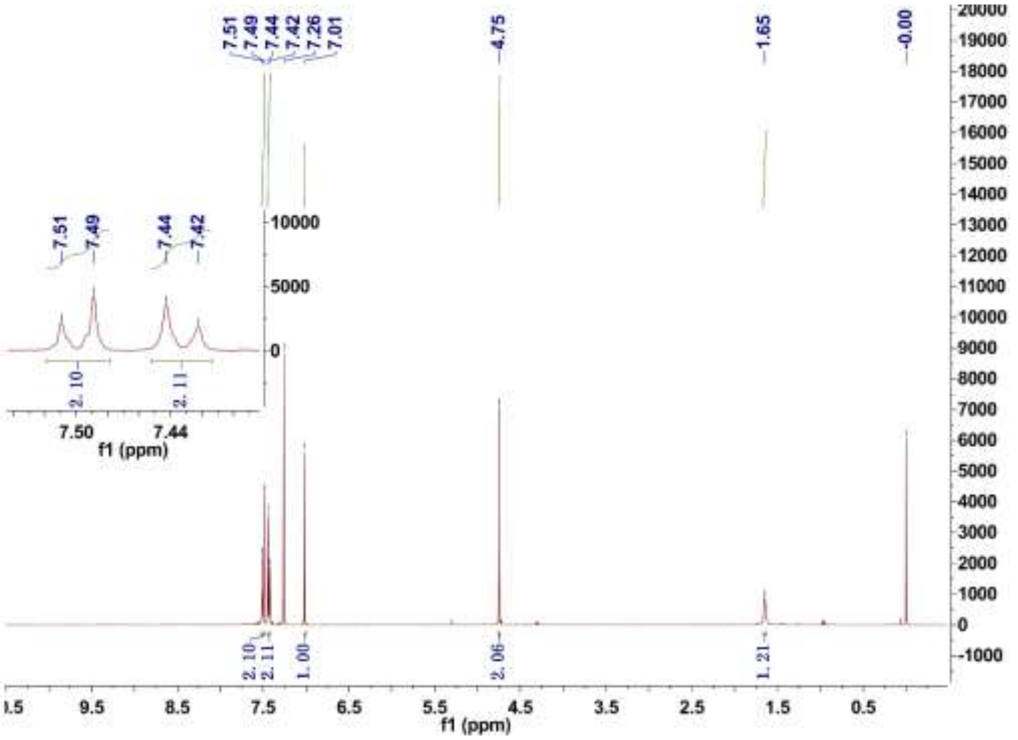

Figure S20. ^{1}H-NMR (400 MHz, $CDCl_3$): Compound 4.

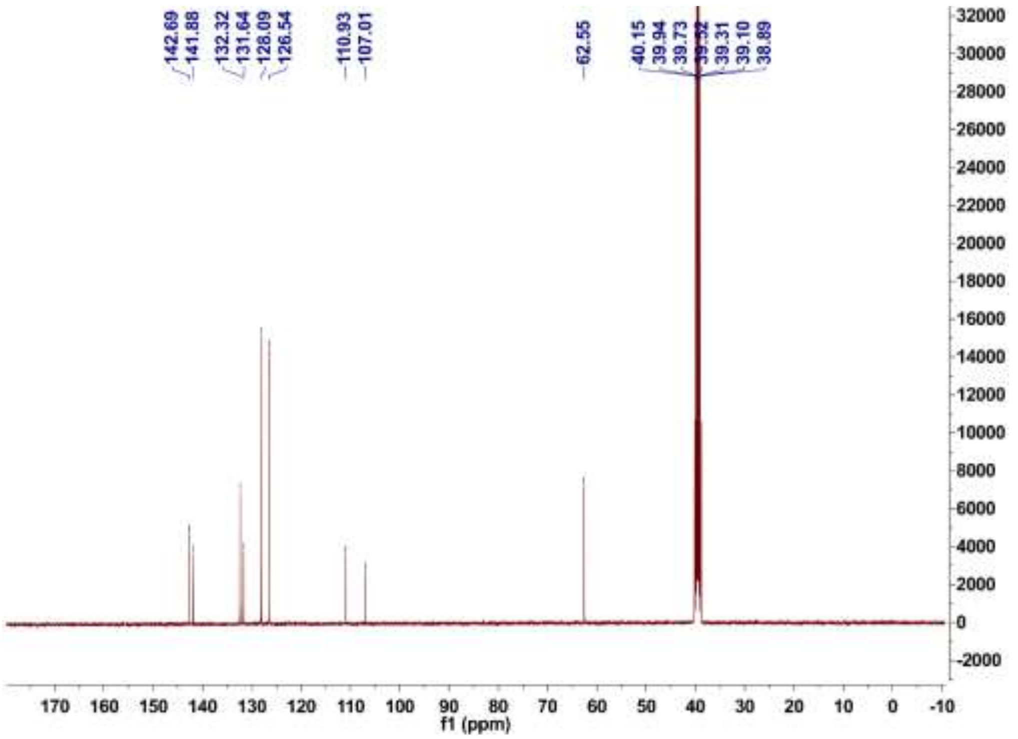

Figure S21. ^{13}C-NMR (100 MHz, DMSO-d_6): Compound 4.

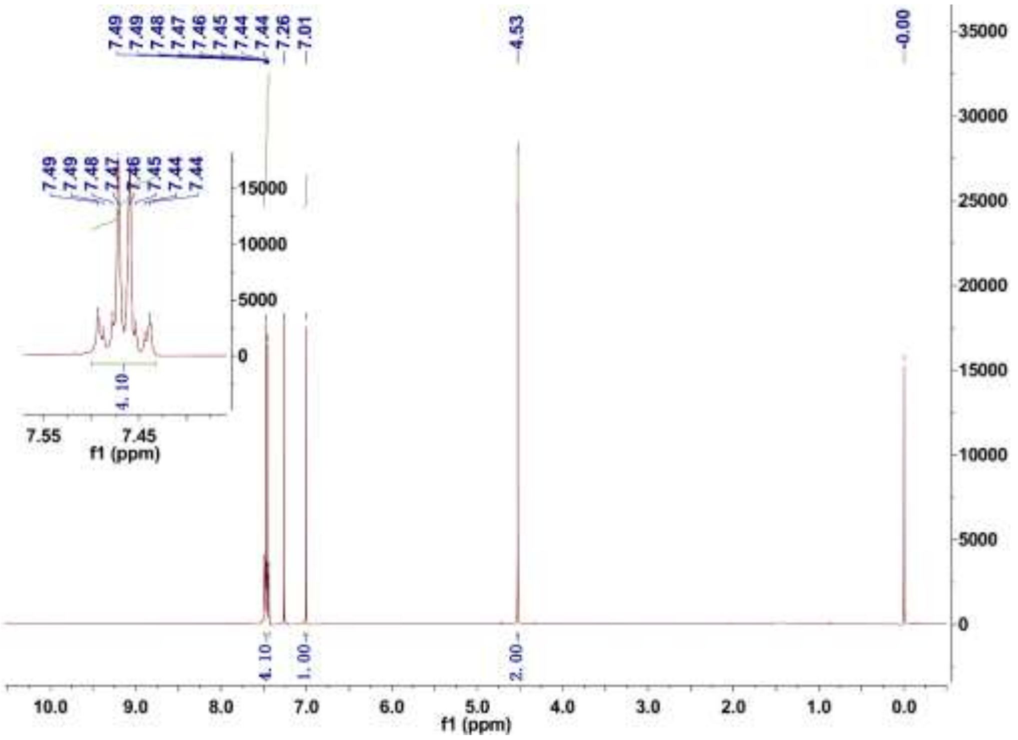

Figure S22. ^{1}H-NMR (400 MHz, $CDCl_3$): Compound 5.

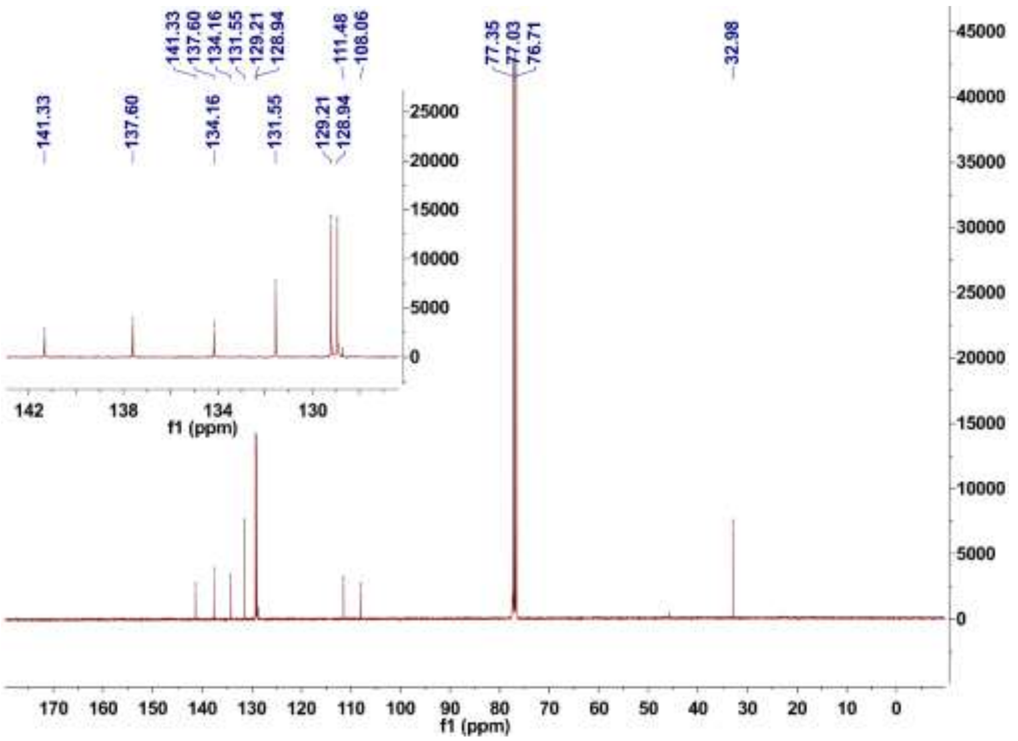

Figure S23. ^{13}C-NMR (100 MHz, $CDCl_3$): Compound 5.

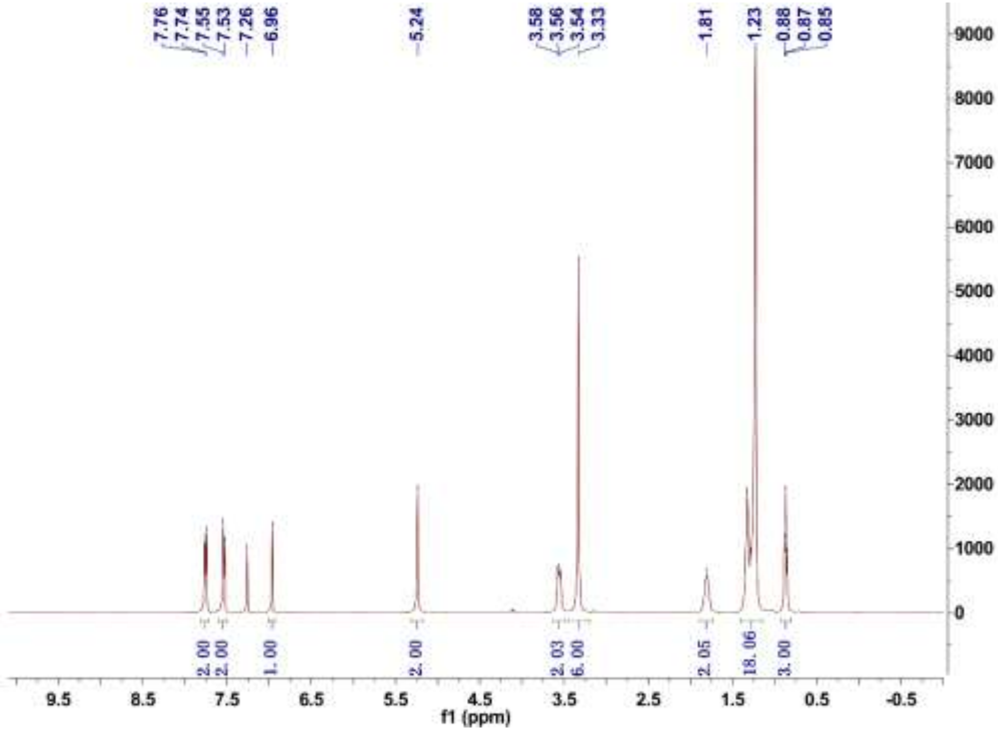

Figure S24. ^{1}H-NMR (400 MHz, $CDCl_3$): Compound 6.

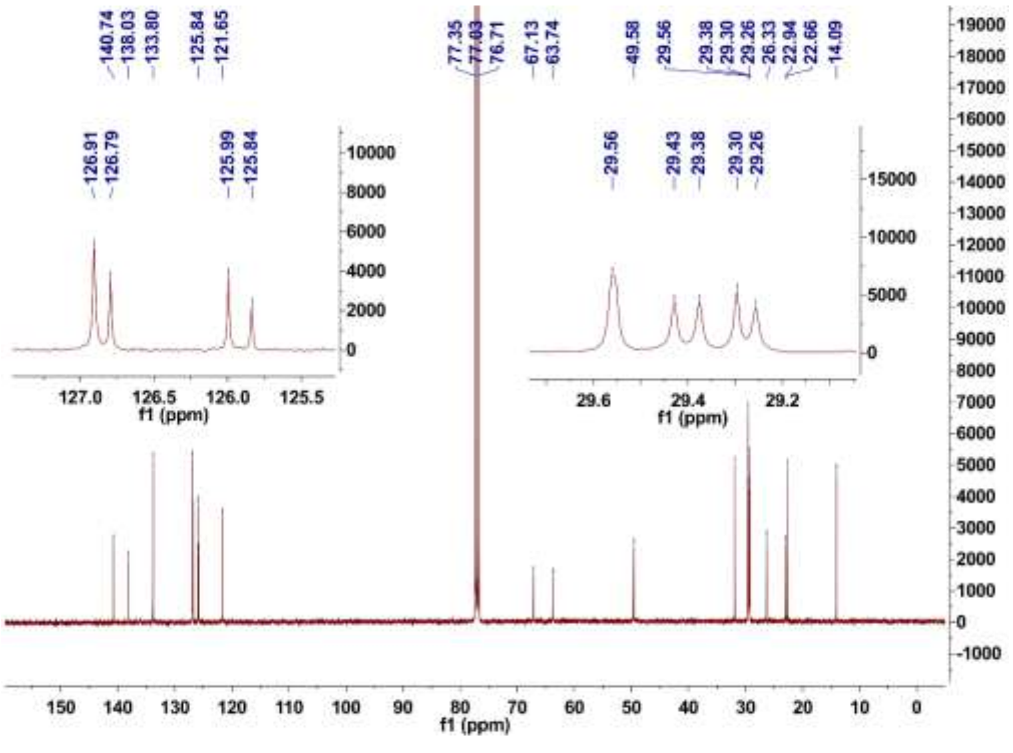

Figure S25. ^{13}C-NMR (100 MHz, $CDCl_3$): Compound 6.
